# The Paths to Negative and Positive Experiences of Informal Caregiving in Severe Mental Illness: A Study of Explanatory Models

**DOI:** 10.3390/ijerph16193530

**Published:** 2019-09-20

**Authors:** Luísa Campos, Carlos Mota Cardoso, João Marques-Teixeira

**Affiliations:** 1 Faculty of Education and Psychology, Universidade Católica Portuguesa, 4169-005 Porto, Portugal; 2 Research Centre for Human Development, Universidade Católica Portuguesa, 4169-005 Porto, Portugal; 3 Faculty of Psychology and Educational Sciences of the University of Porto, R. Alfredo Allen, 4200-135 Porto, Portugal; 4 Laboratory of Neuropsychophysiology, Faculty of Psychology and Educational Sciences of the University of Porto, 4200-135 Porto, Portugal

**Keywords:** experience of caregiving, informal caregivers, severe mental illness, schizophrenia

## Abstract

The experience of caregiving in severe mental illness is a valuable concept for research and clinical practice as it can provide access to the idiosyncratic assessment of negative and positive dimensions of informal caregiving, thus allowing the design of interventions focused on reducing risk factors and promoting protective factors. This study was aimed at testing explanatory models of negative and positive experiences of caregiving considering the role of the caregiver’s perceptions of difficulties, satisfaction, and coping. A convenience sample of 159 informal caregivers of patients with schizophrenia was used in this study. Different variables were considered: (1) perception of difficulties (Caregiver’s Assessment of Difficulties Index); (2) perception of satisfaction (Caregiver’s Assessment of Satisfaction Index); (3) perception of coping (Caregiver’s Assessment of Managing Index); and (4) the experience of caregiving (Experience of Caregiving Inventory). Using structural equation modeling, the results revealed the following: (1) the perception of difficulties and of satisfaction coexist; (2) the negative experiences of caregiving are predominantly explained by the perception of difficulties and of coping with stress; and (3) the positive experiences of caregiving are mainly explained by the perception of sources of intrapersonal satisfaction, while the perception of coping does not have robust predictive value.

## 1. Introduction

### 1.1. From Hospital to Community: Deinstitutionalization and Caregiver Burden

During the second half of the twentieth century, the direction of public mental health policy was to transfer the care of patients from state mental hospitals to settings in the community, with deinstitutionalization policies and the community mental health movement progressively increasing the number of people with chronic and severe mental illness (e.g., schizophrenia) living in their own homes, with hospital services being used only in situations of acute need [1,2]. Although deinstitutionalization was established with the best of intentions, it has resulted in unintended consequences due to insufficient planning to provide alternative care with adequate resources [3]. Thus, on the one hand, this paradigm shift has brought benefits to the families of the mentally ill, in particular, the chance to be closer to the person and take care of them [3]; on the other hand, the passage of patients into the community has resulted in large costs for families including quantifiable costs in time, money and lost opportunities, as well as psychological costs, which are difficult to quantify and scope. It is in this context that research has focused on the concept of family burden (Treudley, 1946), which refers to the negative consequences for those who are in close contact with persons with serious mental illness [4,5].

### 1.2. From Caregiver Burden to the Experience of Caregiving

Over the past decades, the concept of burden has been extensively and thoroughly studied, and a solid body of scientific research has been developed that focuses on the impact of the informal caregiving of people with specifics diagnostics (e.g., Alzheimer’s disease, cancer, and stroke). Within this research field, there is an extensive amount of nursing literature that focuses on the impact of caring for older people [6,7,8,9,10], people with a physical illness [11] or a mental illness. Also, several reference assessment tools have been developed [10]. Specifically, there are several important works regarding the caregiving of persons with severe mental illness, including the impact of mental illness on parents [12,13] and on siblings [14], literature reviews [15,16], and the development of theoretical models [17,18].

Several studies have explored the relationship between burden and its components, both objective and subjective [19], and sociodemographic variables of the patient and the caregiver (e.g., gender, age, academic qualifications, family relationships, living with the patient, being the principal caregiver) and clinical variables of the patient (e.g., diagnosis, symptoms, onset and duration of the illness). Although some consistent relationships have been found (e.g., patient symptomatology, caregiver age, living with the patient), these results should be read with caution, given the limitations of the concept of burden and its evaluation methods (see [4,20,21,22,23,24,25]). As Szmukler and colleagues noted [25], the concept of burden fails to identify the possible positive aspects of caregiving and address determinants, mediating influences, or outcomes. Also, the concept could be an erroneous imputation of normal changes in the illness, and the items of its measures have built-in assumptions that disruptions are caused by the patient, and that these disruptions are the cause of the caregiver’s distress. In addition, methodological limitations have been mentioned in studies focused on the burden concept, such as variability in sample procedures or the heterogeneity of the studied population (see [26]).

In the late 1990s, the concept of the experience of caregiving (EC) emerged [25], as well as a new assessment instrument, the Experience of Caregiving Inventory (ECI) [25] due to the limitations in the concept of burden as suggested by several studies. From this perspective, the study of EC was performed via the stress–appraisal–coping paradigm [27], and was conceptualized in terms of an appraisal of its demands. According to this conceptualization, the appraised stressors -include the patient’s illness, behaviors, and disabilities, and the perceived disruptions to the caregiver’s life, as well as different mediating factors such as the quality of family relationships or the degree of social support, which may influence the appraisal. In this way, both the threat or risk aspects and the positive aspects of the role of the caregiver are taken into account. The result of the appraisal and the caregiver’s coping strategies should be construed in terms of psychological or physical morbidity as well as well-being.

Research has begun to highlight the relevance of the evaluation process and of coping as being crucial to explain the variability in the experience of caregiving [5,25,28,29,30], the exploration of positive dimensions of caregiving [22,25,31,32,33], the impact of gender on caregiving [34], the relationship between demographic and clinical variables in the experience of caring for patients with first-episode psychosis [35], and how caregivers perceive the symptoms and behaviors of their sick family member [30].

Despite the relevance of this concept for both research and clinical practice, research is still scarce.

## 2. Materials and Methods

### 2.1. Present Study

The present study, based on the conceptualization of the EC, is part of larger study [26,36] involving 159 caregivers of people with schizophrenia, that aimed (a) to socio-demographically categorize caregivers and patients; (b) to characterize the EC; (c) to characterize patients’ frequency of symptoms and behaviors as perceived by their caregivers; (d) to identify the psychopathology of caregivers; (e) to characterize the difficulties and degrees of disturbance, possible sources and degrees of satisfaction, and coping strategies and degrees of effectiveness as perceived by caregivers; (f) to study the relationships between the aforementioned variables; and (g) to identify the main explanatory variables of the experience of caregiving. The following results are from this larger study with informal caregivers, which in turn, informed the present secondary analysis.

The larger study revealed that positive and negative experiences are not mutually exclusive; they coexist. Positive and negative perceptions of the experience of caregiving were positively and significantly correlated. Specifically, with regard to the relationship between perception of coping and perception of satisfaction, strong associations were observed between problem-solving strategies and alternative perception strategies and intrapersonal satisfaction and satisfaction related to results, and dealing with stress strategies and interpersonal satisfaction. Perceptions of symptoms and behaviors of patients, psychopathology of caregivers, difficulties, and the negative experience of caregiving were positively and significantly correlated. Perceptions of satisfaction and the positive experience of caregiving were positively and significantly correlated, but there were no associations between these variables and the psychopathology of caregivers or the perception of symptoms and behaviors of patients. Caregivers who perceived greater sources of satisfaction and more positive experiences of caregiving had higher levels of intimacy and cohesion with the patient. Half of the participants had indicators of psychopathology, and women and parents had higher values, even though they had more positive experiences of caregiving. Women also had more strategies to deal with stress and more negative experiences of caregiving [36]. Multiple linear regression models showed that perception of strategies to deal with stress and perceived difficulties explained 60% of the variance in the negative experience of caregiving, and the perception of intrapersonal satisfaction and alternative perception strategies explained 49% of the variance in the positive experience of caregiving. Coping with stress strategies also explained some of this variance, though less significantly [36].

Based on these results, the present study aimed to test explanatory models of negative and positive experiences of caregiving, considering the role of the perceptions of difficulties, satisfaction, and coping. Three working hypotheses were defined.

**Hypothesis** **1.** *Perception of difficulties and perception of satisfaction explain both positive and negative experiences of caregiving.*

**Hypothesis** **2.** *The negative experience of caregiving is explained by the perception of more difficulties and the greater perception of coping with stress.*

**Hypothesis** **3.** *The positive experience of caregiving is explained by the perception of more sources of satisfaction and the perception of coping with problems (problem-solving strategies and alternative perception strategies).*

### 2.2. Participants

We used a convenience sample of 159 informal caregivers (not involved in caring for another person with a mental or physical disorder, older than 17 years) of people with a diagnosis of schizophrenia (of at least 1 year, excluding affective psychoses, older than 17 years, not institutionalized), from the Association of Families of People with Mental Disorders (80%) of northern, central, and southern Portugal, and were between 34 and 70 years of age (median age = 55).

The majority of participants were female (67%), married/unmarried partners (66%), and parents (71%, 50% mothers, 21% fathers); other caregivers were siblings (13%), partners (4%), children, (3%) and other relatives or friends (9%), and most considered themselves as primary caregivers (73%). Concerning their educational and professional status, 57% had a secondary school or higher education, and 60% were at home, unemployed, or retired. Most caregivers reported living with the patient (77%) and having daily contact with them (83%).

Participants were informal caregivers of 134 patients with a diagnosis of schizophrenia (as diagnosed by their specialists), and a median age of 36 years (P10–P90, 23–47). The majority were male (81%), single (85%), with no children (85%), at home/unemployed/retired (86%), and had a secondary school or higher education (57%). Regarding the patients’ mental illness, 81% had been diagnosed for 5 or more years, 96% had psychiatric support and 45% had psychological support. Half of the patients were integrated in socio-occupational forums or socio-professional rehabilitation (54%), and these activities were attended daily by almost all of those patients (90%).

### 2.3. Measures

*Sociodemographic variables of the caregiver*: To characterize the sample, sociodemographic data were collected: gender, age, marital status, education, professional situation, degree of kinship with the patient, if the participant considered him/herself the primary caregiver, if the caregiver lived with the patient, and the frequency of contact with the patient.

*Perception of difficulties*: The Caregiver’s Assessment of Difficulties Index (CADI) [8] (Portuguese translation [37]) is a self-reported questionnaire used to identify the nature of problems faced by caregivers and which ones are seen to be most stressful. It consists of 30 items (e.g., “I no longer have a meaningful relationship with the person I care for”) that ask caregivers whether or not a particular aspect of caregiving is difficult (if not, this is indicated as a score of zero). If they answer in the affirmative, then they are asked to rate the level of stress (1 = never applies, 2 = sometimes applies, 3 = always applies). The total score ranges from 0 to 90. Two content adaptations were made in items 6 and 24 due to the target group of this study. The original item 6, “The person I care for depends on me to move,” was replaced by “The person I care for depends on me in daily activities,” and the original item 24, “The person I care for suffers from incontinence,” was replaced by “The person I care for refuses to do his daily personal hygiene.” In the current sample, Cronbach’s alpha for the global score was 0.944.

*Perception of satisfaction*: The Caregiver’s Assessment of Satisfactions Index (CASI) [8] (Portuguese version [37]) is a self-reported questionnaire used to identify and quantify the perception of caregiving factors as a source of satisfaction. It consists of 30 items (e.g., “I get pleasure from seeing the person I care for happy”) that ask caregivers whether the situation applies to their caregiving situation (if not, this is indicated as a score of zero). If they answer in the affirmative, they evaluate their degree of satisfaction with the situation (1 = no real satisfaction, 2 = quite a lot of satisfaction, 3 = a great deal of satisfaction). Items are organized in three dimensions: interpersonal satisfaction, intrapersonal satisfaction, and satisfaction related to results. Scores range from 0 to 27 for interpersonal satisfaction, 0 to 42 for intrapersonal satisfaction, and 0 and 21 for satisfaction related to results. In the current sample, Cronbach’s alpha for interpersonal satisfaction, intrapersonal satisfaction, and satisfaction related to results was 0.788, 0.894, and 0.748, respectively.

*Perception of coping*: The Caregiver’s Assessment of Managing Index (CAMI) [8] (Portuguese translation [37]) was used to explore caregivers’ subjective experience of the use and efficacy of coping strategies. It consists of 38 coping statements organized in 3 subscales: (1) problem-solving strategies; (2) alternative perception strategies; and (3) dealing with stress strategies. For each statement, caregivers answer based on a 4-point scale: 0 = does not apply, 1 = applies but they do not find it helpful, 2 = applies and they find it quite helpful, or 3 = applies and they find it very helpful. In this study, Cronbach’s alpha was 0.840 for problem-solving strategies, 0.798 for alternative perception strategies, and 0.590 for dealing with stress strategies.

*Experience of caregiving*: The Experience of Caregiving Inventory (ECI) [25] (Portuguese ECI version was developed by Manuel Gonçalves-Pereira and its acceptability and construct validity was reportedin [38]) was used to assess the experience of caregiving. The ECI is a 66-item self-reported questionnaire with 8 negative and 2 positive subscales. The negative subscale includes difficult behaviors (e.g., unpredictable), negative symptoms (e.g., noncommunicative), stigma (e.g., how to explain the illness to others), problems with services (e.g., how health professionals do not take you seriously), effects on the family (e.g., how family members do not understand the illness), loss (e.g., what sort of life the family member might have had), dependency (e.g., being able to do the things you want to do), need for backup (e.g., family member’s difficulty with looking after money). The two positive scales are positive personal outcomes (e.g., I have learned more about myself) and good aspects of the relationship with the patient (e.g., the patient has grown in strength in coping with the illness). The items are rated on a Likert scale from 0 (never) to 4 (nearly always). Scores range from 0 to 208 for the negative subscale and from 0 to 56 for the positive subscale. In the current sample, Cronbach’s alpha for the negative and positive subscales was 0.947 and 0.848, respectively.

The patient’s sociodemographic and clinical variables were also obtained: gender, age, marital status, number of children, education, professional situation, with whom the patient lives, age at which the diagnosis was made, and types and frequency of support.

Finally, other variables were collected for the informal caregivers and patients; however, since this paper presents partial results of a larger study, only the variables related to the objectives of this paper are described.

## 3. Procedure

### 3.1. Data Collection

Data collection followed ethical guidelines, with all participants signing an informed consent form. The sociodemographic form and questionnaire were self-administered by participants, who were almost all involved in the Association of Families of People with Mental Illness.

The study presented in this paper is part of a doctoral thesis approved at the Faculty of Psychology and Educational Sciences of the Universidade do Porto and funded by Fundação para a Ciência e a Tecnologia (SFRH/BD/13508/2003), approved and monitored in accordance with all the requirements demanded for research (Ethics Committee of Faculty of Psychology and Educational Sciences of the University of Porto 2019/07-4).

### 3.2. Analytic Plan

A descriptive analysis was used to characterize the participants in relation to sociodemographic characteristics, the perception of difficulties, satisfaction, and coping, and the experience of caregiving. Discrete variables were described via absolute and relative frequencies (%) and continuous variables were described using medians, 10th and 90th percentiles, averages, and standard deviations.

The scales and subscales of the measures used were calculated according to the indications of the authors of the original versions and their analysis was carried out assuming that these were continuous variables. For a comparison of the results of the scales and subscales (CAMI, CASI, and ECI), which varied in different intervals, standardized values were calculated (z-scores).

Psychometric properties, i.e., internal consistencies in the different measures used were evaluated using Cronbach’s alpha.

To study the hypothesized models, we tested them in accordance with the instructions proposed by experts, which suggest using the following combination of indicators: structural equation modeling (SEM) [39,40] chi-squared (≥0.5) along with its associated probability, root mean square error of approximation (RMSEA, the closer to 0, the better; *p* > 0.5); normed fit index (NFI, ≥0.95 for acceptance); root mean square residual (RMR, the smaller, the better; 0 indicates perfect fit); goodness of fit index (GFI, 1 indicates perfect fit); relative fit index (RFI, the closer to 1, the better); and non-centrality parameter (NCP, confidence interval should be closer).

The following variables were considered: dependent variables: negative and positive experiences of caregiving; and independent variables: perception of difficulties, satisfaction, and coping.

Based on the results reported in Section 2.1, one latent variable was created: perception of coping with problems, which included problem-solving strategies and alternative coping perceptions.

In all hypothesis tests, a level of significance of α = 5% was used.

The analyses were performed using SPSS Statistics^®^ v.17.0 (Statistical Package for the Social Sciences, Chicago, IL, USA) and SPSS Statistics^®^ Amos^TM^ v.6.0 (Statistical Package for the Social Sciences, Chicago, IL, USA).

## 4. Results

Two hypothesized models for the negative experience of caregiving and two for the positive experience of caregiving were developed. For all models, the following relationships were considered (from the results described in Section 2.1):

(1) The influence of interpersonal satisfaction on the perception of difficulties (i.e., the perception of fewer sources of interpersonal satisfaction is associated with a greater perception of difficulties).
(2) The influence of the perception of difficulties on the perception of coping (i.e., the perception of more difficulties is associated with the perception of using more strategies to deal with stress).
(3) Retroactivity between the perception of coping and the perception of satisfaction (i.e., the perception of more satisfaction is associated with the perception of using all coping strategies; the perception of using more strategies to deal with stress is associated with the perception of more intrapersonal satisfaction; and the perception of using more problem-solving strategies and alternative perception strategies is associated with the perception of more intrapersonal satisfaction and satisfaction related to results).

A dichotomy of the perception of coping was defined: coping with problems (problem-solving strategies and alternative perception strategies) and coping with stress (perception of strategies to deal with stress), and all of the sources of perception of satisfaction were considered.

**Figure 1:**
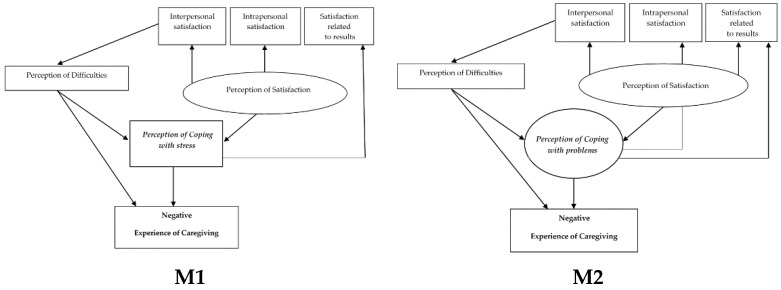
Hypothesized theoretical models for negative experience of caregiving.

For the negative experience of caregiving, two hypothesized models were developed, where the dependent variable is explained by the perception of difficulties and of coping: Model 1 (M1) for coping with stress and Model 2 (M2) for coping with problems (see Figure 1).

**Figure 2:**
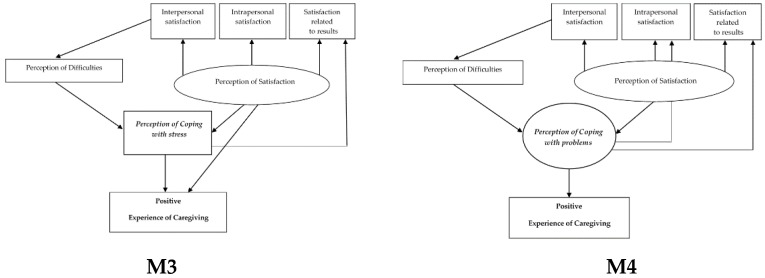
Hypothesized theoretical models for positive experience of caregiving.

For the positive experience of caregiving, two hypothesized models were developed where the dependent variable is explained by the perception of satisfaction and of coping: Model 3 (M3) for the perception of coping with stress and Model 4 (M4) for the perception of coping with problems (see Figure 2).

The suitability of the measurement models was tested, with the results indicating good adjustment for M1 and M2 (although M1 had better results), reasonable adjustment for M3, and poor for M4 (see Table 1).

The acceptance of any model is determined by the combination of all of its indicators, with experts recommending against taking any one value as a single reference for accepting or rejecting the model [41]. Therefore, and according to the values of the chi-square adjustment tests and the adjustment indicators, for the negative experience of caregiving, M1 presented the best adjustment, and for the positive experience of caregiving, M3 presented the best adjustment.

**Figure 3:**
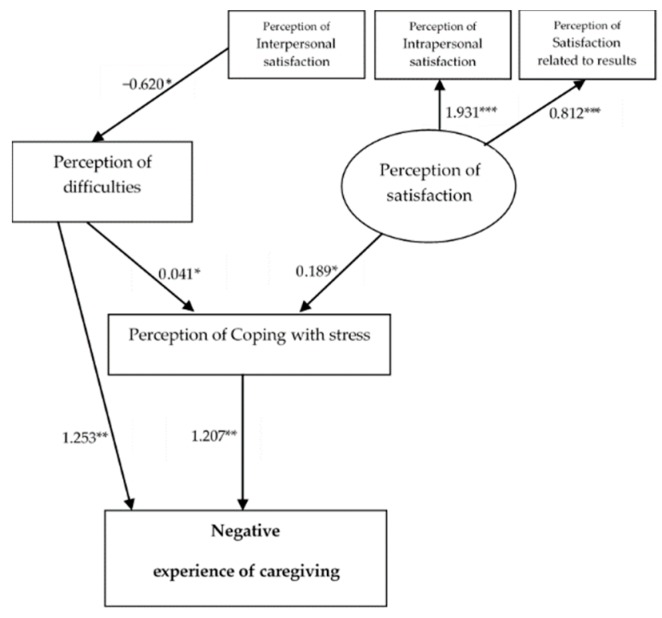
Standardized solutions of the accepted M1 model. Note: Only considered the significant estimates of regressive coefficients; * *p* < 0.05; ** *p* < 0.01; *** *p* < 0.001.

**Figure 4:**
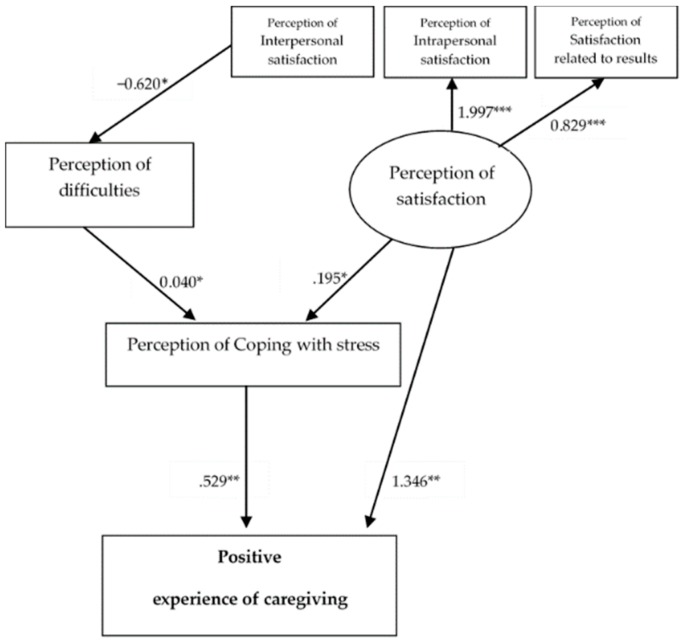
Standardized solutions of the accepted M3 model. Note: Only considered the significant estimates of regressive coefficients. * *p* < 0.05; ** *p* < 0.01; *** *p* < 0.001.

Regarding the effects observed between the independent variables studied, an individual analysis of the regression coefficients of the final models of negative and positive experiences of caregiving (Figure 3 and Figure 4) revealed that almost all of the proposed pathways reached the level of significance (*p* < 0.05).

Both models had (1) negative and statistically significant regression coefficients between perception of interpersonal satisfaction and perception of difficulties; (2) low positive and statistically significant regression coefficients between latent variable perception of satisfaction and perception of coping with stress; (3) low positive and statistically significant regression coefficients between perception of difficulties and perception of coping with stress; and (4) high positive and statistically significant regression coefficients between the latent perception of satisfaction and the observed variables (interpersonal, intrapersonal, and satisfaction related to results) as the highest value in both models, with intrapersonal satisfaction (see Figure 3 and Figure 4).

Regarding the regressive coefficients of M1, high and statistically significant positive coefficients were found between perception of coping with stress and perception of difficulties and the negative experience of caregiving.

With regard to M3, statistically significant positive coefficients between perception of coping with stress and perception of satisfaction and the positive experience of caregiving were found, and the latter relationship was more significant.

## 5. Discussion

The present study involved 159 informal caregivers of patients with schizophrenia and was aimed at testing explanatory models of negative and positive experiences of caregiving, considering the roles of the perception of difficulties, sources of satisfaction, and coping.

As hypothesized, the accepted models indicated that positive and negative experiences of caregiving follow parallel paths and coexist in both models, with perception of difficulties and sources of satisfaction being a predominant part of the negative experience, explained by the perception of difficulties and the perception of coping with stress. However, the hypothesis concerning the positive experience of caregiving was only partially confirmed, since it was mainly explained by the perception of sources of intrapersonal satisfaction, while the perception of coping did not have robust predictive value.

While perception of difficulties and perception of satisfaction are the main explanatory variables of the negative and positive experiences of caregiving in the proposed models, the discussion of the results should first consider the relationships between the dependent variables: perception of difficulties, satisfaction, and coping. These relationships will be discussed for both models, since the results were similar.

### 5.1. Coexistence of Perception of Difficulties and Sources of Satisfaction in the Experience of Caregiving

First, both models included perception of difficulties and perception of satisfaction, reinforcing the suitability of this concept: caring for a person with severe mental illness could include the perception of threat as well as the perception of positive aspects [25,31,42,43,44,45]. Furthermore, the sources of perception of interpersonal satisfaction are the benefits derived from the relationship between the caregiver and the patient, for themselves, the patient, or both, and may be associated with a positive relationship between caregiver and patient, underlining the idea suggested by different researchers, for example in [8], that positive relationships in caregiving contribute to caregiver satisfaction. The sources of interpersonal satisfaction seem to be coping resources that buffer the difficulties of caregiving [8].

### 5.2. Role of Perception of Intrapersonal Satisfaction in the Experience of Caregiving

Second, in both models, perception of satisfaction was predominantly determined by the perception of intrapersonal satisfaction. The sources of satisfaction include the caregiver’s ability to recognize that they are performing their obligations; they are useful and esteemed because of the caregiving; they are able to develop new skills and overcome obstacles because of the caregiving; they have developed and matured as individuals because of the caregiving; and finally, the caregiving gave them a sense of life that they did not have otherwise. These results reinforce data from the literature that emphasize that sources of intrapersonal satisfaction are the most important positive dimensions of caregiving in mental illness. This will be discussed further in the specific discussion of the model for the positive experience of caregiving.

### 5.3. Role of Perception of Coping with Stress in the Experience of Caregiving

Third, the two models had positive and statistically significant regressive coefficients between perception of difficulties and satisfaction with and perception of coping with stress. This result may be because the sample was composed of caregivers of people diagnosed with a chronic disease, and when a situation is assessed as nonmodifiable, as in this case, coping that focuses on dealing with stress is most commonly used [46].

### 5.4. Path to a Negative Experience of Caregiving

The negative experience of caregiving can be explained by a greater perception of coping with stress and the perception of more difficulties, an expected result that reinforces the previous regressive models (see Section 2.1). Regarding the role of perception of difficulties, although it can be hypothesized that there may be important convergent validity between the instruments (CADI and negative dimension of ECI), this should also be understood within the scope of the results described in Section 2.1. In the larger study, positive and significant associations between “negative” variables not considered in the structural equation modeling were found: caregiver’s psychopathology, patient’s perception of symptoms and behaviors, perception of difficulties and the negative experience of caregiving, and perception of dealing with stress strategies. These variables may have an important role in the perception of difficulties and the negative experience of caregiving. The robust explanatory value of the perception of coping with stress in the negative dimension of the caregiving experience is understood by the explanatory value of the perception of difficulties, which is inevitably associated with a greater activation of coping strategies, specifically those that allow greater relief from the tension.

### 5.5. Path to a Positive Experience of Caregiving

Concerning the positive experience of caregiving, it was hypothesized that this would be explained by the perception of more sources of satisfaction and the perception of coping with problems. This hypothesis was partially confirmed. The proposed model for the positive experience of caregiving was mainly explained by the perception of sources of intrapersonal satisfaction, while the perception of coping (with stress) did not have robust predictive value. As with the negative experience of caregiving model, it can be hypothesized that there may be important convergent validity between the instruments (CASI and positive dimension of ECI), although results indicate that the positive dimension of caregiving seems to mainly result from intrapersonal sources of satisfaction. These sources arise from caregivers fulfilling their caregiving role and duty and the meaning they attribute to the caregiving (meaning of life, maturation, growth, development of new skills, self-esteem), as previously mentioned. For this discussion, it is important to refer to the results in Section 2.1, where it was reported that the participants in this sample who perceived more positive experiences from caregiving were female and parents.

Concerning gender differences, there is a relatively small amount of research data with inconclusive results. However, it can be hypothesized that women caregivers provide care differently and therefore have a different experience compared to men. Studies by Carol Gilligan [47] revealed that women appear to have different psychological and moral tendencies than men. Gilligan (1993) [47] argued that women’s moral orientation is focused predominantly on caring and concern for others, while men’s moral orientation emphasizes the abstract principles of justice. It is possible that women, with a moral orientation focused on care will develop differential caregiving. This differentiation allows them to better capture positive experiences that arise from the caregiving. Nolan and colleagues (1996) [8] reported that the nutritional aspects of caregiving are more likely to be satisfying for women than men. In addition, Gonçalves Pereira (1996) [48] reported that the capacity of acceptance has been a secular stereotype of women. In this sense, the ability to accept, which is probably related to having a moral orientation focused on care may allow a greater perception of positive experiences since there is a greater ability to accept illness and better understand the patient. The interaction of all these aspects can lead to greater personal growth, more maturity, a more positive relationship with the patient, and finally, a greater appreciation of the outcomes.

Regarding the type of kinship, a study developed by Schwartz and Gidron (2002) [33] explored the positive aspects and benefits of caregiving and found that all parents considered that their perception of satisfaction depended mainly on feeling fulfilled by parental tasks and learning about themselves. The authors noted that the extent to which parents perceived the experience of caregiving as rewarding was a function of how they assessed their roles and responsibilities in the situation, that is, how they created “meaning.” The same researchers, relying on Frankl (1984), pointed out that one of three suggested paths to discovering the meaning of life would be one’s attitude toward inevitable suffering. In this sense, caring for a child with mental illness becomes a concrete path on which parents can discover and create meaning and purpose in their lives and caregiving could be perceived as a process of self-actualization, “a basic path for growth, for the whole, for the fulfilment” [33] (p. 152). These meanings are deeply linked to the relationship with the patient, the roles per se (e.g., being the patient’s father/mother) and their meaning, and the types of caregiving per se (e.g., living with the patient) and their meaning (differential care). This path to positive experiences explains that coping does not have robust predictive value in this dimension, contrary to its role in the negative experience of caregiving model.

To sum up, and integrating the data obtained in SEM with those of the large study in which this work was developed, on the one hand, the path to negative experiences seems to be predominantly determined by “negative” variables. On the other hand, on the path to positive experiences, the psychopathology of the caregiver and the perception of more symptoms in the patient do not seem to have impact. The main variables involved are the perception of greater sources of intrapersonal satisfaction, being a parent, being a woman, and having a positive relationship with the patient.

For professionals involved in caregiving support, our study reinforces the need to work not only to alleviate their difficulties, but also to promote the positive dimension of caregiving. Results from the present study reinforce the need to systematically include in interventions:

(1) The intentional identification and promotion of the positive dimensions of caregiving. Thus, in these interventions, health professionals should promote awareness of the positive dimension, helping caregivers to identify aspects such as their role in promoting patient well-being, the possible existence of personal growth, possible maturation, the sense of life, and the sense of role fulfillment. Promoting the existence and identifying possible sources of satisfaction will enable the construction of the meaning of caregiving, which will buffer difficulties, and also strengthen self-awareness, self-esteem, self-confidence, crucial dimensions to promote the empowerment of the caregiver. This work should be systematic and intentionally done since according to Schwartz and Gidron [33], most caregivers seem to give little or no consideration to the positive potential of caregiving until they are requested/asked to comment on it. Furthermore, as suggested by previous research [49], for the effective accomplishment of this work it is necessary that professionals follow up on perceived joy.
(2) The intentional promotion of relationships between caregiver and relative with severe mental illness, helping to preserve relationships where they are in good order, improving them where they are disturbed, and stimulating them where growth is still possible.

Interventions should be designed taking into account the specificities of each family and the idiosyncrasies of the experience of caregiving, given the factors that are involved. The constant monitoring of the defined plans is essential so that the intervention is adjusted to the needs. Healthcare policy should systematically consider this broader approach in order to prevent the growth of a new group of patients: family caregivers. Specialized professionals, such as mental health nurses and psychologists are in a key position to provide this support.

In future studies, some of the limitations of the present study can be addressed, namely, those concerning the integration of sociodemographic (gender and type of kinship) and clinical (psychopathology) variables of the caregiver and relational variables (quality of the relationships between the caregiver and the patient and other family members) in explanatory models of the experience of caregiving that can help us toward a deeper understanding of this complex experience. Regarding the negative experience of caregiving, it will be important to explore the role of the psychopathology of caregivers and the perception of the patient’s symptoms and behaviors. Specific to psychopathology, it is important to understand whether this variable is predominantly a mediator or a result of individual experience, since the stress–appraisal–coping model suggested that there is a reversibility of antecedents, mediators, and outcomes, and at different times in the flow of events, “an antecedent may become a result, and vice versa” [50] (p. 142). With respect to future models of positive experiences of caregiving, the exploration of gender roles and types of kinship of caregivers and the quality of patient relationships should be considered. In addition, it will also be important to use qualitative methods, such as interviews, that allow a deeper and idiosyncratic understanding of the relationships between these different variables.

Finally, it is relevant to test the differential effectiveness of interventions focused on burden and the experience of caregiving, comparing their impacts on psychological and physical morbidity as well as well-being.

## 6. Conclusions

This study tested explanatory models of negative and positive experiences of caregiving in severe mental illness using structural equation modeling. Two paths were proposed: the negative experience was explained by the perception of difficulties and the perception of coping with stress, and the positive experience was mainly explained by the perception of sources of intrapersonal satisfaction, while the perception of coping did not have robust predictive value. In addition, the presence of the perception of difficulties and of satisfaction in both experiences and the role of interpersonal satisfaction in buffering the difficulties experienced by caregivers were highlighted.

These results reinforce the usefulness of the concept of the experience of caregiving, as well as the need to take into account the main areas and content related to possible sources of satisfaction and explore these with caregivers in order to develop interventions by different health professionals such as psychologists and nurses, that not only focus on risk factors and difficulties but also promote positive factors. When caring for someone with severe mental illness, along with perceived difficulties, there may be satisfaction; and this satisfaction plays an important role in buffering difficulties. Both the satisfaction that stems from interpersonal relationships, and the satisfaction that comes from the “inside” of the caregivers themselves allow for the construction of the meaning of caregiving. Interventions should fit with these two types of satisfaction.

## Figures and Tables

**Table 1:** Goodness-of-fit indicators for the hypothesized theoretical models.

Model	X^2^ (df)	*p* (X^2^)	RMSEA (CI)	*p* (RMSEA)	RMR	GFI	NFI	RFI
M1	7.199 (6)	0.303	0.036 (000–0.114)	0.533	7.533	0.985	0.985	0.964
M2	14.705 (9)	0.099	0.063 (0.000–0.120)	0.308	6.350	0.975	0.977	0.946
M3	11.333 (6)	0.079	0.075 (000–0.141)	0.227	4.162	0.978	0.975	0.937
M4	19.685 (9)	0.020	0.087 (0.033–0.139)	0.112	5.833	0.966	0.968	0.924

RMSEA, root mean square error of approximation; CI, confidence interval; RMR, root mean square residual; GFI, goodness of fit index; NFI, normed fit index; RFI, relative fit index.
